# The *bla*_NDM-1_-Carrying IncA/C_2_ Plasmid Underlies Structural Alterations and Cointegrate Formation *In Vivo*

**DOI:** 10.1128/AAC.00380-19

**Published:** 2019-07-25

**Authors:** Sead Hadziabdic, Jennie Fischer, Maria Borowiak, Burkhard Malorny, Katharina Juraschek, Annemarie Kaesbohrer, Beatriz Guerra, Carlus Deneke, Bruno Gonzalez-Zorn, Istvan Szabo

**Affiliations:** a Department for Biological Safety, German Federal Institute for Risk Assessment (BfR), Berlin, Germany; b European Food Safety Authority, Parma, Italy; c Departamento de Sanidad Animal and Centro de Vigilancia Sanitaria Veterinaria, Facultad de Veterinaria, Universidad Complutense de Madrid, Madrid, Spain

**Keywords:** NDM-1 carbapenemases, *Salmonella*, broiler chicken infection study

## Abstract

In 2012, a carbapenemase-producing Salmonella enterica serovar Corvallis isolate carrying a *bla*_NDM-1_ multiresistance IncA/C_2_ plasmid, apart from IncHI2 and ColE-like plasmids, was detected in a wild bird in Germany. In a recent broiler chicken infection study, we observed transfer of this *bla*_NDM-1_-carrying IncA/C_2_ plasmid to other Enterobacteriaceae. Here, we focused on the stability of this plasmid and gained insight into the type and frequency of its structural alterations after an *in vivo* passage in a broiler chicken infection study.

## Abstract (precis)

In 2012, a carbapenemase-producing Salmonella enterica serovar Corvallis isolate carrying a *bla*_NDM-1_ multiresistance IncA/C_2_ plasmid, apart from IncHI2 and ColE-like plasmids, was detected in a wild bird in Germany. In a recent broiler chicken infection study, we observed transfer of this *bla*_NDM-1_-carrying IncA/C_2_ plasmid to other Enterobacteriaceae.

## INTRODUCTION

Antimicrobial resistance is described as the most urgent threat to global public health and food safety today (1). In a previous broiler chicken infection study, we demonstrated that the multidrug-resistance *bla*_NDM-1_-carrying IncA/C_2_ plasmid (pRH-1238) is transferable, without antibiotic pressure, to intestinal Escherichia coli strains and a Klebsiella pneumoniae strain (2). In this *in vivo* study, we aimed to investigate and understand the dynamic of structural alterations in the *bla*_NDM-1_-carrying IncA/C_2_ plasmid pSE12-01783-2 with Salmonella enterica serovar Corvallis as host. We evaluated if these alterations are sporadic or frequently occurring events and whether they influence further *in vitro* transfer. With the use of Illumina and Nanopore whole-genome sequencing (WGS) analysis, we aimed to detect structural alterations occurring in a *bla*_NDM-1_-carrying pSE12-01783-2 plasmid and reveal the full structure of the cointegrated *bla*_NDM-1_-carrying IncHI2-IncA/C_2_ megaplasmid.

## 

### Broiler chicken infection study.

**FIG 1:**
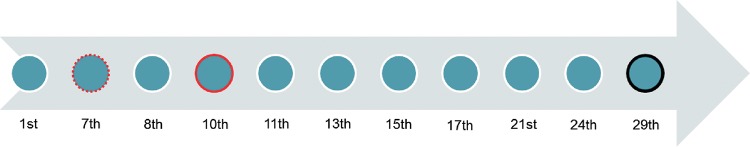
Experimental design containing sampling days (blue circles); recipient [*S*. Paratyphi B (*d*Ta+), *S*. Enteritidis, and *S*. Infantis] inoculation in groups 2, 3, and 4 (red dotted outer ring) on 7th day of life; and donor (*S*. Corvallis) inoculation in groups 1 to 4 (red solid outer ring) on 10th day of life. The black outer ring indicates the end of the experiment with cecum removal postmortem at the 29th day of life.

Each experimental group (G1 to G4), consisting of 10 1-day-old Ross 308 broiler chicks (T1 to T10), was placed separately in the Facilities for Animal Experimentation at the German Federal Institute for Risk Assessment. The experimental design is shown in Fig. 1. Challenge strains were inoculated orally, with inoculum containing ∼5 × 10^6^ CFU of the respective challenge strain in 100 μl. Animal experiments were approved by the German State Authority for Health and Social Affairs (Lageso) (no. 0308/15).

### Donor and recipient strains.

As a model strain, an avian native *Salmonella* Corvallis (GenBank accession number CP027677) strain carrying three plasmids, 284,485-bp IncHI2 (pSE12-01738-1, CP027678), multiresistance NDM-1-encoding 177,190-bp IncA/C_2_ (pSE12-01738-2, CP027679), and 10,047-bp ColE-like ColRNAI (pSE12-01738-3, CP027680), was selected (3). As potential recipients of the *bla*_NDM-1_-carrying pSE12-01738-2 plasmid, nalidixic acid (NAL)-resistant avian native Salmonella enterica serovar Paratyphi B (*d*Ta+) (13-01617), Salmonella enterica serovar Enteritidis (07-03428), and Salmonella enterica serovar Infantis (14-03263) were selected. This investigation describes structural alterations and not transfer of the *bla*_NDM-1_-carrying IncA/C_2_ pSE12-01738-2 plasmid *in vivo*. For the study of genome and plasmid content alteration among *S*. Corvallis isolates, reisolates from three chicks belonging to four groups were selected. The selection of strains is shown in Table 1. To evaluate the *in vivo* stability of the plasmid content without selective pressure in isolation, 7 *S*. Corvallis reisolates detected on xylose lysine deoxycholate (XLD) agar from different chicks of group 1 were included. These are shown in Fig. S2 in the supplemental material. In total, 97 *S*. Corvallis reisolates were characterized in depth for the purpose of this investigation.

**TABLE 1:**
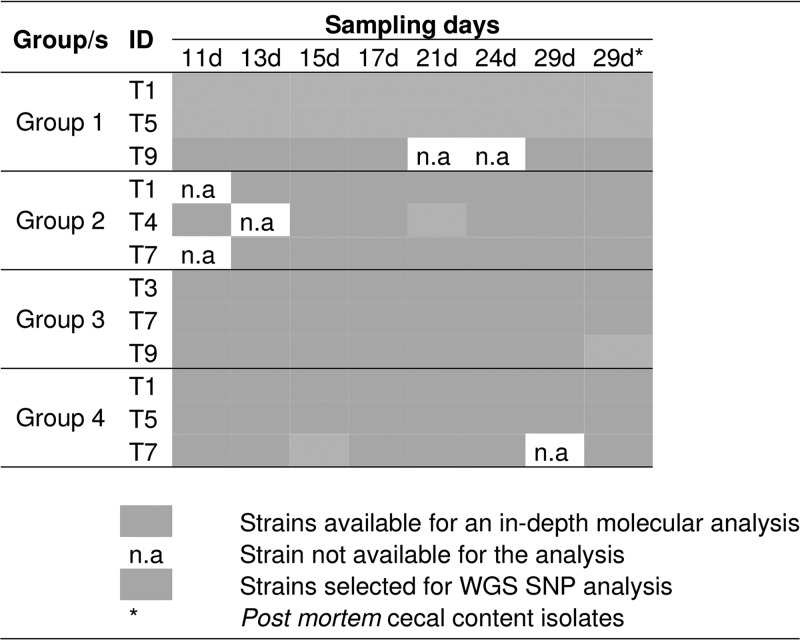
Distribution of 90 *S*. Corvallis strains which were selected for in-depth molecular analysis, including the strains isolated from the cecal content (29d*)^*a*^

a Darker gray, strains selected for in-depth molecular analysis (all isolates, except n.a.); lighter gray, strains selected for WGS SNP analysis (see “Whole-genome SNP analysis” paragraph below). n.a, strain not available for analysis; *, postmortem cecal content isolates.

### Isolation of *S*. Corvallis reisolates.

*S*. Corvallis reisolates were isolated by suspending fresh fecal droppings from each chick in 4.5 ml of 0.85% (wt/vol) NaCl, from which a 100-μl deposition volume was plated in duplicates onto XLD plates (Thermo Fisher Scientific, Germany) supplemented with 1 mg/liter cefotaxime (CTX) and 0.125 mg/liter meropenem (MEM). The addition of cefotaxime as a second antibiotic was to inhibit overgrowth by *Pseudomonas* spp. with intrinsic carbapenem resistance, which could hamper detection and quantification of the donor strain. Seven *S*. Corvallis strains from group 1 were isolated by plating onto XLD without selective supplementation. Strains were preserved at −80°C for later molecular analysis. Prior to molecular analysis, strains were serotyped. The designation is based on group (G1 to G4), day of isolation (1st to 29th day of life), and chick identifier (ID) (T1 to T10).

### S1-PFGE plasmid profiling and *bla*_NDM-1_ hybridization of *S*. Corvallis reisolates.

All 97 reisolates of *S*. Corvallis were subjected to S1 pulsed-field gel electrophoresis (PFGE). Generated fragments were separated by the CHEF-DRIII system (Bio-Rad Laboratories, Spain) under running conditions as previously described (4). The S1-PFGE gels of strains from group 1 and group 2 were further used for Southern blotting and *bla*_NDM-1_ hybridization.

### *In vitro* conjugation experiments.

Filter mating conjugation experiments with *S*. Corvallis strains harboring pSE12-01738-2 variants (D1, D2, D3, and D4) from the *in vivo* trial were conducted. Plasmid profiles of these are shown in Fig. S1 in the supplemental material. As recipients, nalidixic acid-resistant *S*. Paratyphi B (*d*Ta+), *S*. Enteritidis, and *S*. Infantis were selected. From overnight cultures of selected strains, 500 μl was inoculated into 25 ml (1:50) of Luria Bertani Bouillon-Miller liquid (LBL) and grown aerobically at 37°C with shaking (200 rpm), until optical density at 600 nm (OD_600_) reached a value of 0.2. Afterward, donor and recipient strains were mixed 1:2 (100 μl:200 μl) and centrifuged (16,000 rpm for 5 min). Here, 200 μl of supernatant was discarded and the pellet was resuspended in the remaining 100 μl and plated on an 0.22-μm-pore-size mixed-cellulose ester membrane filter (Merck Millipore, Germany) placed on LB agar (Thermo Fisher Scientific, Germany). Conjugation experiments lasted 4 h and were repeated at room temperature (RT), 37°C, and 41.5°C. NDM-1-producing *Salmonella* transconjugants were selected on XLD containing 1 mg/liter CTX, 0.125 mg/liter MEM, and 50 mg/liter nalidixic acid (NAL) and confirmed by serotyping, and conjugal transfer frequency (CTF) was calculated per donor.

### WGS analysis.

The whole-genome sequencing (WGS) analysis was conducted with Illumina MiSeq technology. Strains were grown overnight at 37°C in 4 ml of LBL with 1 mg/liter CTX, from which 1 ml was processed for DNA extraction using the PureLink genomic DNA minikit (Invitrogen, USA). DNA concentration (ng/μl) was measured with the Qubit fluorometric quantitation (Invitrogen) system. Sequencing libraries were prepared with the Nextera XT DNA sample preparation kit (Illumina, San Diego, CA, USA). Paired-end sequencing was performed with the Illumina MiSeq benchtop (MiSeq Reagent v3 600-cycle kit, 2 × 251 cycles). Raw reads were assembled *de novo* using CLC Genomics Workbench 9.5.2 (Qiagen, Hilden, Germany), and sequence types (STs), plasmid types, and resistance genes were detected using BatchUpload (5). Comparison of the pSE12-01738-2 variants (D3 and D4) was performed by mapping the raw reads to the reference pSE12-01738-2 plasmid (GenBank accession number CP027679) and visualizing them using BRIG (6).

### Oxford Nanopore MinION sequencing.

The *S*. Corvallis reisolate G2-21d-T4 (D2) carrying an ∼450-kb cointegrate of pSE12-01738-1 IncHI2 and pSE12-01738-2 IncA/C_2_ plasmid was sequenced with MinION technology. The sequencing library was prepared from genomic DNA using the Rapid Sequencing kit (Oxford Nanopore Technologies, Oxford, United Kingdom) and sequenced for approximately 16 h using the Flow-MIN106 R9 flow cell.

For genome assembly, the hybrid assembly software Unicycler (v0.4.4) was used (7). It starts from an initial SPAdes short-read assembly and simplifies the assembly using information from short and long reads, thereby achieving a complete and accurate assembly (8). Assemblies were polished using Pilon (9). The cointegrated megaplasmid is represented using CLC Genomics Workbench 9.5.2.

### Whole-genome SNP analysis.

Reisolates from chicks T1 (*n* = 8) and T5 (*n* = 8) in group 1 were selected. Three *S*. Corvallis reisolates (*n* = 3) carrying variants of pSE12-01738-2 (D2, D3, and D4) were also included (Table 1). Single-nucleotide polymorphism (SNP) analysis was performed using BioNumerics 7.6 (Applied Maths, Ghent, Belgium). Sequencing raw data were trimmed and mapped against the reference chromosome of *S*. Corvallis 12-01738 (GenBank accession number CP027677 ). To reconfirm SNP position in encoding genes, trimmed reads were mapped also to the annotated reference chromosome of *S*. Corvallis.

### Statistical analysis.

For comparison of CTFs among donors (D1 to D4) under different temperature conditions (room temperature [RT], 37°C, and 41.5°C), statistical analysis with SPSS (ver. 21.0; SPSS Inc., USA) was performed. The distribution of the CTFs is presented by box-whisker plots with outliers and extreme outliers included. For the determination of statistical significance, one-way analysis of variance (ANOVA) was performed and least significant difference (LSD) was used as a *post hoc* test. The differences were considered significant if the *P* value was <0.05.

Plasmid contents of 90 *S*. Corvallis reisolates detected after selective isolation are shown in Table 2, whereas seven *S*. Corvallis reisolates detected without selective isolation are shown in Fig. S2 in the supplemental material. Variation in plasmid content was mainly seen in the loss of the IncHI2 plasmid (31 from 97 selected reisolates). Two reisolates harbored, besides the <10-kb ColE-like (ColRNAI) plasmid, an ∼450-kb megaplasmid (G2-21d-T4 [further marked as D2]) and G1-13d-T9 (shown in Fig. S2 but not included in further *in vitro* analysis). In relation to the *bla*_NDM-1_-carrying IncA/C_2_ pSE12-01738-2 plasmid, ∼70-kb (G3-29d-T9 postmortem [D3]) and ∼10-kb (G4-15d-T7 [D4]) deletions were observed.

**TABLE 2:**
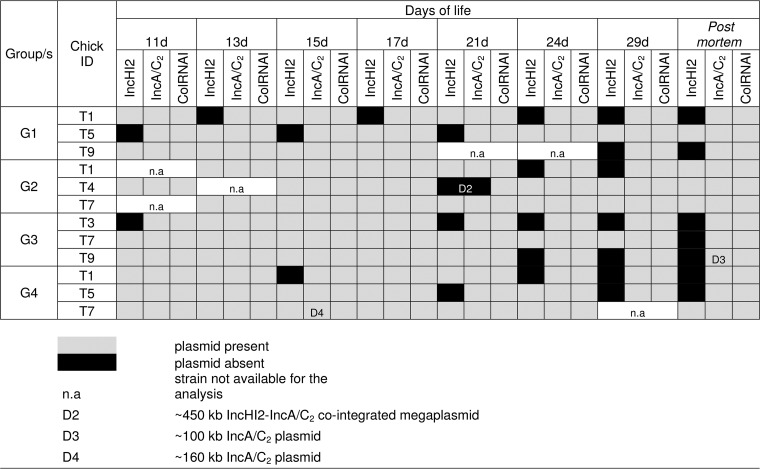
Overview of plasmid content based on S1-PFGE for 90 selected *S*. Corvallis reisolates in groups 1 to 4, indicating complete plasmid loss and strains carrying pSE12-01738-2 variants^*a*^

a Light gray shading, plasmid present; black shading, plasmid absent; n.a, strain not available for the analysis; D2, ∼450-kb IncHI2-IncA/C_2_ cointegrated megaplasmid; D3, ∼100-kb IncA/C_2_ plasmid; D4, ∼160-kb IncA/C_2_ plasmid.

**FIG 2:**
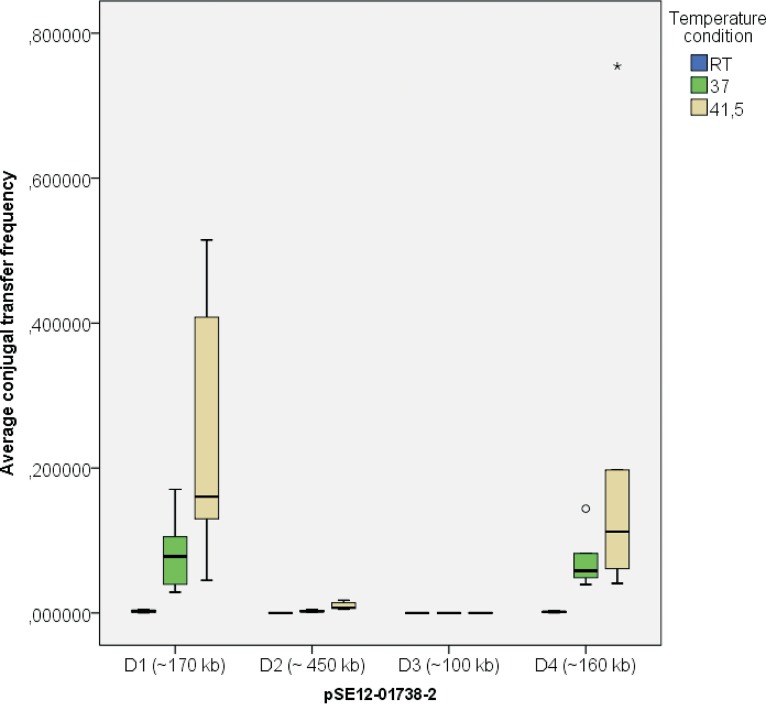
Distribution of the CTF rates for donors (D1 to D4) carrying pSE12-01738-2 variants in relation to three *Salmonella* recipients under different temperature conditions (room temperature [RT], 37°C, and 41.5°C) with outliers (°) and extreme outliers (*) included.

Figure 2 shows distributions of *in vitro* CTF rates for donors (D1 to D4) under different temperature conditions in relation to three *Salmonella* recipients. Among conjugative pSE12-01738-2 variants, the CTFs were highest at 41.5°C. The ∼100-kb pSE12-01738-2 variant D3 was not transferable (Fig. 2). The donor with the ∼450-kb IncHI2-IncA/C_2_ cointegrate (D2) had a statistically significant lower CTF, in contrast to D1 with unaltered pSE12-01738-2 and D4 with an ∼160-kb pSE12-01738-2 plasmid under all three temperature conditions (Fig. 2; see also Table S1).

Following *in vitro* experiments, we observed variation in colony size and prolonged growth of *Salmonella* transconjugants after conjugation with *S*. Corvallis carrying the ∼450-kb cointegrated megaplasmid. Therefore, 24 of these transconjugants (four small and four large colonies per recipient) from conjugation experiments at 41.5°C were analyzed by S1-PFGE. Analysis revealed that the size of the colony is not linked to full ∼450-kb cointegrate acquisition. Additionally, resolution of the cointegrate (plasmids from ∼170 to ∼350 kb in size) in transconjugants was observed (data not shown).

**FIG 3:**
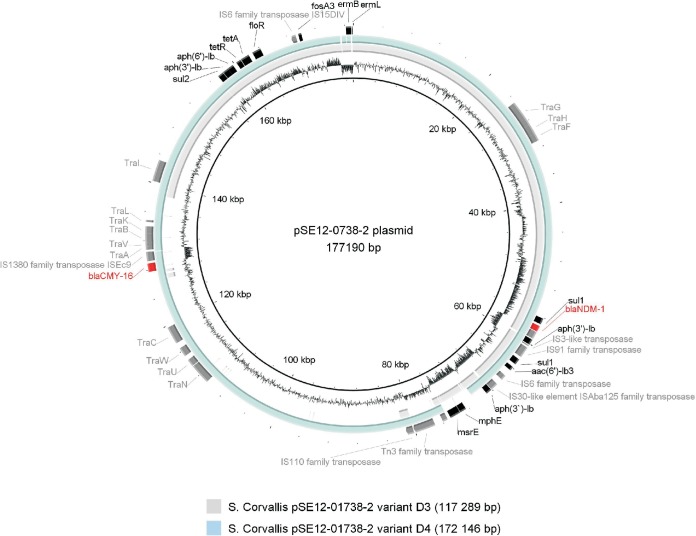
Visualization of the *bla*_NDM-1_-carrying pSE12-01738-2 variants D3 and D4 compared to PacBio RSII reference sequence of pSE12-01738-2 plasmid (GenBank accession number CP027679) using BRIG (6) with resistance genes (red, beta-lactam genes; black, other resistance genes) as well as IS elements, transposase, and *tra* genes (all marked gray).

**FIG 4:**
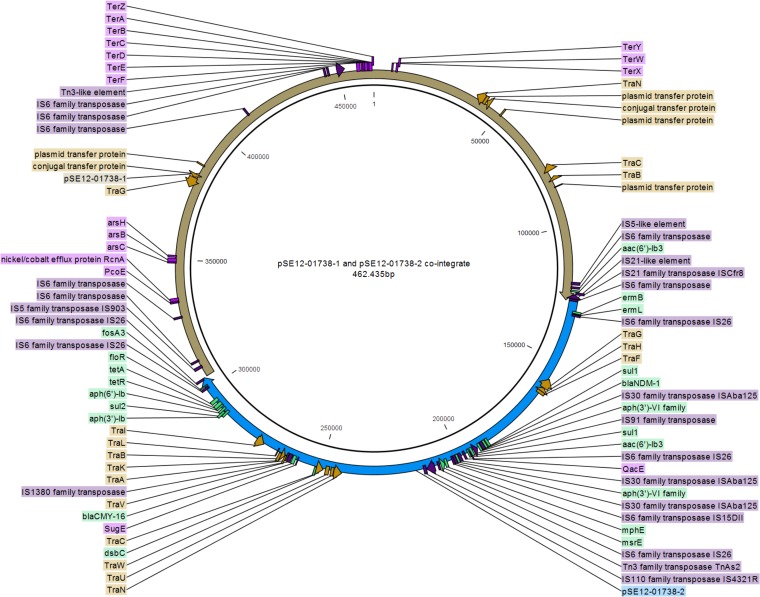
Structure of newly emerged IncHI2 pSE12-01738-1 and IncA/C_2_ pSE12-01738-2 cointegrated megaplasmid (462,435 bp). The fusion of IncHI2 pSE12-01738-1 (brown) and IncA/C_2_ pSE12-01738-2 plasmid (blue) is shown. Resistance genes are marked green, heavy metal resistance genes are pink, transposase and IS elements are purple, and transfer (*tra*) genes are brown.

The WGS analysis revealed that pSE12-01738-2 variant D3 has a consensus sequence size of 117,289 bp and the pSE12-01738-2 variant D4 has a size of 172,146 bp, in contrast to the 177,190-bp pSE12-01738-2 reference plasmid (GenBank accession number CP027679) (Fig. 3). The D2 variant of the pSE12-01739-2 plasmid is a cointegrated megaplasmid (462,435 bp) of pSE12-01738-1 IncHI2 and pSE12-01738-2 IncA/C_2_ (Fig. 4). In three (G1-11d-T1 [position 978935, G→A], G1-29d-T5 [position 2226323, A→G], and G1-29d*-T5 [position 4307016, A→T]) out of 16 selected *S*. Corvallis reisolates from group 1, nonsynonymous polymorphic nucleotide exchanges (SNPs) were observed. These were attributed to genes encoding citrate lyase subunit alpha (G1-11d-T1), dihydroxy-acid dehydratase (G1-29d-T5), and galactitol-1-phosphate 5-dehydrogenase (G1-29d*-T5). In *S*. Corvallis strains harboring variants of the pSE12-01738-2 plasmid, SNPs were not detected.

Structural alterations of the *bla*_NDM-1_-carrying pSE12-01738-2 plasmid were seen in ∼10-kb and ∼70-kb deletion and ∼450-kb megaplasmid formation. The ∼450-kb megaplasmid (462,435 bp) is a cointegrate of IncHI2 (pSE12-01738-1) and the multiresistance *bla*_NDM-1_-carrying IncA/C_2_ (pSE12-01738-2) plasmid (Fig. 4) and was detected in 2 out of 97 strains. The fusion was mediated by IS*6*-like family genetic elements. In a study of movement of IS*26*, which can be identical to IS*6,* it was observed that IS*26* can form cointegrates between DNA molecules (10). Other studies have shown plasticity of IncHI2 and fusion with IncF plasmids (11, 12). A fusion event can potentially facilitate dissemination of other genetic elements, such as heavy metal resistance in the case of tellurite (*Ter* cluster) present in pSE12-01738-1 (Fig. 4). A study by Lin et al. (13) revealed that spread of the *bla*_CTX-M-17_ gene present on a nonconjugative plasmid was due to fusion with a conjugative ∼73-kb plasmid. As our IncHI2-IncA/C_2_ cointegrate was detected in only two reisolates, we assume that such an *S*. Corvallis population persists *in vivo* but in lower numbers. This could be due to instability of the cointegrate, supported by our *in vitro* conjugation experiments where resolution of the IncHI2-IncA/C_2_ cointegrate was observed. Plasmid resolution was observed by Xie et al. (14) in the case of the ∼190-kb cointegrated multireplicon *bla*_NDM-5_ plasmid, suggesting plasmid instability in new recipients or during conjugation. Besides instability and decreased CTF effect, our cointegrate acquisition caused an elongated growth time for *Salmonella* recipients and variation of the colony size *in vitro*. In a study on the transmission and burden of an ∼1-Mb Pseudomonas syringae megaplasmid, pMPP1a107, a decrease in fitness was also observed (15). In another study, it was observed that the same plasmid can have up to 2.5-fold-higher fitness costs in different *Pseudomonas* species (16).

Recently, Paskova et al. (17) detected a *bla*_NDM-1_-carrying ∼300-kb multireplicon (IncA/C_2_ and IncR) plasmid in an E. coli strain from human urine. The type I IncA/C_2_ sequence part of this megaplasmid was 99% identical to pRH-1238, which is the same plasmid as pSE12-01738-2, with only a minor structural deletion in the latter (3). These findings confirmed our hypothesis of the broad host range and adaptation potential of this particular *bla*_NDM-1_-carrying plasmid *in vivo*. This also suggests possible *bla*_NDM-1_ spillover from human clinical settings where carbapenems are an alternative to cephalosporin in cases of resistance (18).

We observed frequent loss of the IncHI2 pSE12-01738-1 plasmid, despite genes associated with the toxin-antitoxin system being present. Plasmids are undergoing selection pressure, and to control costs and maximize their spread, the host adapts strategies to cope with their presence (19). The cost of the pSE12-01738-1 plasmid might have outweighed the benefits for the host, leading to the plasmid loss (20, 21). Structural alterations were more common in IncA/C_2_ pSE12-01738-2 than in the pSE12-01738-1 and pSE12-01738-3 plasmids. These were seen in two deletion events of the pSE12-01738-2 plasmid. The first, smaller deletion (∼10 kb) covers IS*6* family transposase-flanked macrolide resistance genes (*mphE* and *msrE*), and a larger deletion (∼70 kb) included two *tra* clusters (*traL-traK-traB-traV-traA* and *traC-traW-traU-traN*) (Fig. 3). As *tra* genes are required for pilus assembly (*traW*), the structure of pilus (*traC*), and mating pair stabilization (*traN*) (22–24), the absence of some *tra* genes led to loss of the conjugation machinery in this pSE12-01738-2 variant (D3) (Fig. 2 and 3). The remaining *traG*, *traH*, *traF*, and *traI* genes did not maintain conjugation ability for this plasmid derivative *in vitro*.

Antimicrobial usage is the most common trigger for the spread of antimicrobial resistance (25); however, reducing antibiotic use alone is not sufficient to reverse resistance (26). Eliminating antimicrobial selection pressure alone does not lead to plasmid loss in all plasmid-host combinations (27). This was observed in our *in vivo* study. Therefore, insights into mechanisms which trigger and enhance plasmid loss might be an effective addition to support current knowledge as future intervention measures.

Our study revealed the most common structural alterations of a public-health-relevant *bla*_NDM-1_-carrying IncA/C_2_ plasmid once carried with *S*. Corvallis into a broiler flock. Despite structural alterations and plasmid cointegration, the *bla*_NDM-1_ gene is maintained in different IncA/C_2_ variants. For the future, synergy of reduction in antimicrobial usage and alternative approaches, such as promoting plasmid loss, might be an additional contribution aiming to slow the spread of resistance.

## Supplementary Material

Supplemental file 1
