# Supplementary material for: The blaNDM-1-Carrying IncA/C2 Plasmid Underlies Structural Alterations and Cointegrate Formation In Vivo
Source: Antimicrob Agents Chemother. 2019 Jul 25;63(8):e00380-19. doi: 10.1128/AAC.00380-19 (PMC6658791; doi:10.1128/AAC.00380-19)
Supplement: Supplemental file 1 [file AAC.00380-19-s0001.pdf]

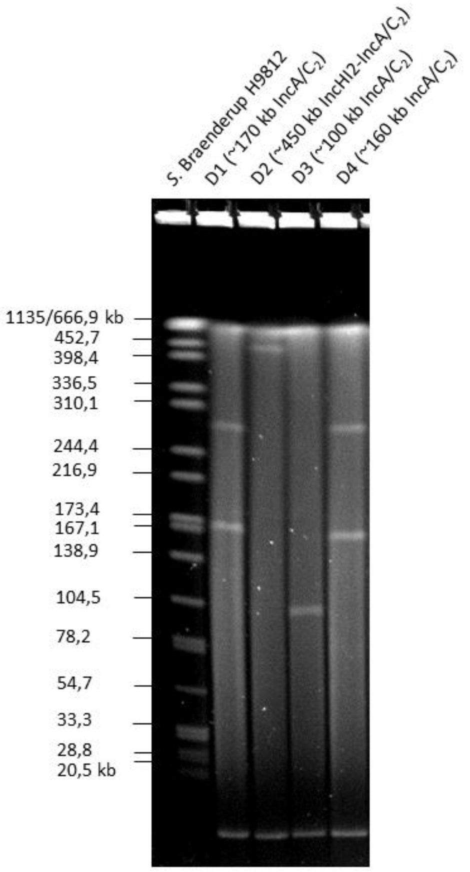

FIGURE S1. The S1-PFGE gel of *S*. Corvallis re-isolates selected as donors (D1-D4) for *in vitro* conjugation experiments. In lane 2 *S*. Corvallis (D1) with unaltered plasmid content. In lanes 3-5 variants of the IncA/C_2 pSE12-01738-2 plasmid; D2 (~450 kb IncA/C_2 pSE12-01738-2 co-integrate), D3 (~100 kb IncA/C_2 pSE12-01738-2) and D4 (~160 kb IncA/C_2 pSE12-01738-2 plasmid). As size marker in lane 1, *S*. Braenderup H9812 (restricted with XbaI).

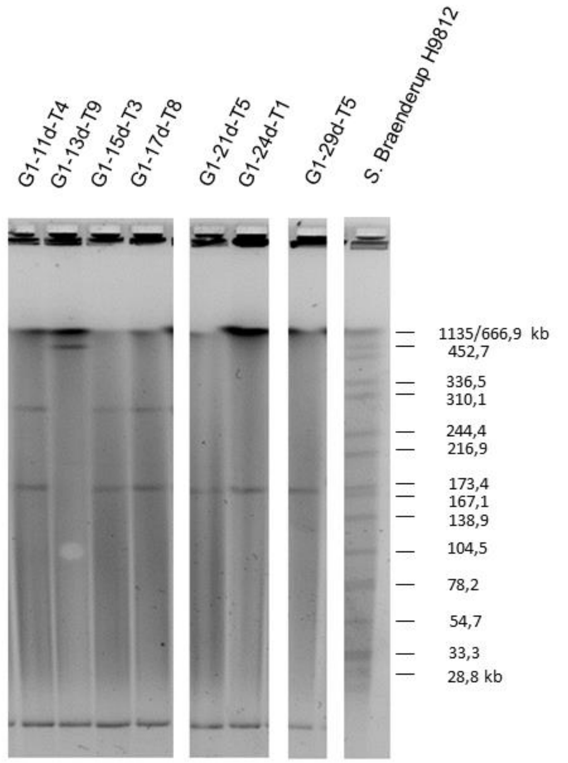

FIGURE S2. S1-PFGE gel of 7 *S*. Corvallis re-isolates from Group 1 (G1) detected on XLD. As size marker in lane 8, *S*. Braenderup H9812 (restricted with XbaI).

TABLE S1. Calculated P-values of CTFs among pSE12-01738-2 variants (D1-D4) based on one-way analysis of variance (ANOVA) and LSD as Post-hoc-Test

	Room temperature (RT)				37°C				41,5°C			
	D1	D2	D3	D4	D1	D2	D3	D4	D1	D2	D3	D4
D1		0,001	0,000	0,158		0,000	0,000	0,537		0,026	0,021	0,806
D2	0,001		0,955	0,015	0,000		0,886	0,001	0,026		0,921	0,044
D3	0,000	0,955		0,013	0,000	0,886		0,001	0,021	0,921		0,036
D4	0,158	0,015	0,013		0,537	0,001	0,001		0,806	0,044	0,036	
